# Connecting and linking neurocognitive, digital phenotyping, physiologic, psychophysical, neuroimaging, genomic, & sensor data with survey data

**DOI:** 10.1140/epjds/s13688-021-00264-z

**Published:** 2021-02-12

**Authors:** Charles E. Knott, Stephen Gomori, Mai Ngyuen, Susan Pedrazzani, Sridevi Sattaluri, Frank Mierzwa, Kim Chantala

**Affiliations:** Social, Statistical, and Environmental Sciences, RTI International, Research Triangle Park, NC USA

**Keywords:** Big data, Wearable technologies, Neurocognitive, Physiological, Passive data, Digital phenotyping, Psychophysical, Data-driven, Systems, Interconnections, Linkage

## Abstract

Combining survey data with alternative data sources (e.g., wearable technology, apps, physiological, ecological monitoring, genomic, neurocognitive assessments, brain imaging, and psychophysical data) to paint a complete biobehavioral picture of trauma patients comes with many complex system challenges and solutions. Starting in emergency departments and incorporating these diverse, broad, and separate data streams presents technical, operational, and logistical challenges but allows for a greater scientific understanding of the long-term effects of trauma. Our manuscript describes incorporating and prospectively linking these multi-dimensional big data elements into a clinical, observational study at US emergency departments with the goal to understand, prevent, and predict adverse posttraumatic neuropsychiatric sequelae (APNS) that affects over 40 million Americans annually. We outline key data-driven system challenges and solutions and investigate eligibility considerations, compliance, and response rate outcomes incorporating these diverse “big data” measures using integrated data-driven cross-discipline system architecture.

## Introduction

Health survey data collection is becoming more complex as researchers demand baseline survey items and other elements like adaptive sampling, continuous physiologic monitoring via wearable technologies, continuous passive digital phenotyping, flash surveys/audio recordings, and genomic data collection. At the same time, response rates are declining while concerns about privacy are growing [1–3]. These forces are all at play in the collection of data from emergency department (ED) trauma patients. Operationalizing this complex and extensive data collection effort requires an integrated and tailored data-driven web-based Information Management System (IMS). In addition to connecting big data sources with baseline health data, the IMS must also control the data flow, allow and enable system interconnections and linkages, enrich compliance prospectively, and adhere to safety, security, and human subject regulations while being user-friendly for participants and clinical and laboratory-based researchers.

We discuss advantages and lessons learned in building data-driven scientific interconnections to collect, enrich, and utilize surveys with various big data sources. In addition, we outline interim eligibility, compliance, and response rates by age and sex in this targeted population when incorporating these state-of-the-art assessments into biomedical research.

## Case study overview

The *AURORA* Study [4] (i.e., *A*dvancing *U*nderstanding of *R*ec*O*very afte*R* Traum*A* [2017 to present with data collection continuing]): Posttraumatic stress, depression, chronic regional and widespread pain, or traumatic brain injury symptoms are common among veterans deployed since September 11, 2001, and are also common among civilian trauma survivors. The National Institutes of Health, in response to an executive order from then-President Obama to initiate major research efforts to better understand and treat these disorders, has funded the most comprehensive longitudinal study of trauma survivors ever performed in the United States.

The AURORA Study Population: Unfortunately from a public health perspective, AURORA’s study population is large given the number of traumatic events that annually occur in the United States. Of the 140 million Americans seen in US EDs [4, 5], more than one-third (over 40 million Americans) present to US EDs for evaluation after trauma exposure [4, 6], and the vast majority of the subset 40 million are discharged home after evaluation and only 10% are hospitalized [4, 6]. Although most of these individuals recover, an important subset develops adverse posttraumatic neuropsychiatric sequelae (APNS). The AURORA cooperative agreement targets the third group, or the ED traumatic event patients discharged home after evaluation, as the sample of interest. However, AURORA also recruits a small subsample of patients from those who are hospitalized to increase the external validity of findings and to facilitate comparison with other major studies that focus exclusively on patients who were hospitalized after ED evaluation [4].

The goal of the AURORA cooperative agreement is to understand, prevent, and predict posttraumatic APNS. Although it is beyond the scope of this manuscript to describe detailed analytic plans [4] for the ongoing cooperative agreement, below we list our scientific aims to help frame this manuscript’s goal of connecting and linking these diverse, broad, and separate data streams, including cutting-edge sources of healthcare big data:

- Aim 1a: Identify/characterize common, discrete, homogeneous APNS using or building on the Research Domain Criteria framework.
- Aim 1b: Identify the most common multidimensional outcomes experienced by trauma survivors.
- Aim 2: Test specific hypotheses regarding the influence of specific pretrauma, trauma-related, and recovery-related factors on the discrete and multidimensional APNS.
- Aim 3: Develop tiered clinical decision support algorithms for multidimensional APNS outcomes, using ensemble machine learning methods and the range of biobehavioral study data collected.

Trauma survivors are enrolled in the immediate aftermath of trauma and followed longitudinally for 1 year. Sophisticated adaptive sampling methods are used to perform a comprehensive, state-of-the-art assessment of genomic, neuroimaging, physiologic, neurocognitive, psychophysical, behavioral, and self-report markers. The study plans to enroll 5000 participants from EDs and will prospectively follow various subsamples for up to 1 year. Prior to the COVID-19 pandemic, our original plan was to recruit approximately 1667 participants/year for 3 years to meet the target goal.

## Literature review

While this case study focuses on connecting and linking diverse big data health elements into the AURORA cooperative agreement, let us step back from a conceptional perspective. Mehta and Pandit [7] performed a systematic review of big data analytics and healthcare. They outline healthcare big data across (1) definitions and concepts, (2) sources, (3) analytical techniques and technologies, (4) benefits and applications, and (5) strategies for overcoming challenges. Most germane to this manuscript are the different definitions and concepts and data sources. AURORA clearly meets the various definitions of healthcare big data across the five Vs: volume, variety, velocity, veracity, and value. We also meet other common big data definitions of energy and life-span [8], types of healthcare data [9], and analytic challenges [8, 10–13]. AURORA encompasses many of the data sources [7, 14] typically referenced as part of healthcare big data: (1) clinical and medical (electronic medical records, diagnostic, prescription, brain imaging, functional magnetic resonance imaging, ancillary); (2) patient-generated (phenotypic, survey, audio recordings); (3) sensor and technology platforms (Verily Study Watch^TM^, digital phenotyping whereby participants use their own smartphones via Mindstrong Discovery APP^TM^, neurocognitive assessments via TestMyBrain^TM^ web-based technology); and (4) genomic (DNA, RNA, and plasma via blood specimens; saliva).

Other recent healthcare big data manuscripts focus on the medical internet of things [15] and summarize the technologies involved and on the internet of things network for healthcare [16] theoretical constructs such as topologies, architectural and physical elements standards, platform, services, and applications at a broader population-based level, recognizing the numerous challenges in the medical internet of things/internet of things network for healthcare. Other manuscripts address interoperability standards and ontologies concerning the web of things [17]. These articles summarize the current state of big data healthcare at a broad level across technologies, standards or lack of them and the inherent challenges in ingestion and harmonization given the diversity of divergent systems and platforms involved. In terms of our AURORA case study, these publications are academic guideposts. However, our manuscript discusses specifics of connecting and linking healthcare big data in the context of a complex biomedical study that had to balance these issues with a tight timeline, critical protocol specifications, regulations and contractual terms and conditions, and specific data use agreements (DUAs). System access, functionality, privacy, confidentiality, The Health Insurance Portability and Accountability Act of 1996 (HIPAA), and producing high-quality and scientifically defensible data generation served as the RTI IMS guardrails.

## Data-driven design & system parameters

Requirements like budget, timelines, and needed approvals (such as DUAs, security, privacy, and confidentiality) directly influence system specifications, design, and ability to achieve complex scientific data collection, processing, linkage, and analytic objectives. When budgets are restricted or timelines are tight, the ability to integrate IMS data with the primary survey/phenotypic tools is critical. The IMS allows for and integrates using wearables (i.e., Verily [Google] Study Watch^TM^), smartphone data collection via digital phenotyping, neurocognitive assessments, biobehavioral data, and genomic data with survey data to achieve scientific aims and objectives. Given the magnitude of concurrent data collection across 30–40 EDs and participants being enrolled daily over a 4- to 5-year period, we needed to factor that having 5000 separate Study Watches was unrealistic and not needed for scientific objectives. Integration allows for adaptive sampling, immediate data access and sharing between surveys, and external data collections apps and tools while being cost efficient. To meet these challenges, RTI International has developed flexible and configurable systems with interoperable goals such as an internal study management system (Nirvana) [18] and survey authoring tools (Hatteras) [19] that allow for immediate sharing of case management data with newly collected survey data for sophisticated study management system purposes. They were also architected for customizations to meet studies’ unique needs, establishing new accounts with apps for launching ecological/wearable technologies, neurocognitive assessments, mobile digital phenotyping, and other data sources to create a biobehavioral health data warehouse.

The existing Nirvana and Hatteras tools utilize an SQL Server as the backend database and allow for language harmonization. Each tool implements security features such as production encryption (i.e., at collection, rest, and transfer) to account for personally identifying information and protected health information in health studies. The integration of these security features simplifies the process and saves significant time in adhering to various regulations—e.g., the Privacy Act [20], HIPAA [21]—and makes it easier to establish Business Associate Agreements (BAA [22]). These cost efficiencies enable customizations of data-driven approaches to digitize the IMS and the protocol electronic flow as opposed to creating one-off systems. We would not have been able to meet the time and budget constraints when developing this IMS without having a base system that already had 65% of the database designed and functional.

### Research protocol acknowledgement

The majority of the discussions in this paper concern the AURORA Study, a national initiative to improve the understanding, prevention, and treatment of APNS [4]. AURORA is a National Institutes of Health (NIH) and private foundation–funded cooperative agreement with Dr. Samuel McLean, contact principal investigator, at the University of North Carolina at Chapel Hill, School of Medicine (UNC). Multiple principal investigators include Dr. Ronald Kessler, Harvard Medical School; Kerry Ressler, McLean Hospital; and Karestan Koenan, Harvard University.

Trauma survivors, ages 18–75, are enrolled through EDs in the immediate aftermath of trauma. Over the following year, participants engage in a state-of-the-art battery of self-report, physiologic, neurocognitive, digital phenotyping, neuroimaging, psychophysical, and genomic assessments. Data collection includes web-based surveys, neurocognitive assessments, ecological monitoring via a smartphone app and via a Study Watch, biospecimens, and for a subsample, in-person deep phenotyping assessments, including functional magnetic resonance imaging. A maximum of 1250 separate data collection events and reminders are currently scheduled for each participant over the study year. The number of data collection events has fluctuated per Institutional Review Board (IRB)-approved amendments throughout the course of multi-site and -year cooperative agreement and is dependent on the adaptive sampling schema/algorithms.

### Regulatory requirements & approvals

RTI International deferred to UNC’s IRB per the NIH’s IRB policy [23]. Our staff provided training credentials for UNC’s IRB records. Because RTI designed and created and now hosts, maintains, and owns the IMS, we placed the IMS in our HIPAA-compliant secure computing center and executed a BAA with UNC.

### Data collection schema

The data schema (Table 1) incorporates multiple external sources of big data generation and associated intellectual property and patents. Below, we outline the complex, prospective data collection schema using (1) phenotypic variables across nine multi-mode surveys, (2) ecological monitoring via the Study Watch, and (3) physiologic, biologic, neurocognitive, symptom, and health outcome assessments across multiple times points over the 1-year participation period.

**Table 1:** AURORA data collection schema [4]

Assessment type	ED	W1	W2	W3	W4	W8	W12	M3	M6	M9	M12
Self-Report	x		x			x		x	x		x
Flash Surveys	x	Daily	Every other day during W3 to W12					Weekly rotating assessments			
Passive Digital	Continuous (every day for a year)										
Study Watch (aka Wearable)	Continuous (every day)							Variable ✓□^1^ (weekly or daily)			
Neurocognitive Assessment	x	Day 2 and then Weekly rotating battery						Quarterly rotating battery			
Neuroimaging			x^1^						x^1^		
Psychophysical			x^1^						x^1^		
Medical Record	x										
Blood	x								x^1^		
Saliva	x^1^	x^1^	x^1^	x^1^	x^1^						

The combination of data collections involving survey and big data streams. ED = emergency department (i.e., baseline data); W = Weekly data; and M = Monthly data. ^1^Subsample of participants via adaptive sampling.

Because of this complexity, separate DUAs were required for Verily’s Life Sciences Study Watch™ and Mindstrong Health’s digital phenotyping app™. These are complicating factors that may impact data environment, system architecture and technologies used, contractual terms and conditions, and quality assurance and quality control systematic processes.

### Identification numbers & linkage

Connecting non-survey data sources to phenotypic data requires the ability to link each system’s records together correctly. Resources (time, in particular), security, and the degree of system integration were important considerations in developing the links between the systems. Table 2 lists the approach used across the varied data sources. Note the above comment about DUAs and implications on security, monitoring, and policies.

**Table 2:** Illustrative big data linkage identification scheme

ID no.	Description	Format
IMS Nirvana	Internal SQL unique number at the individual record *(only resides at RTI)*	####
PID	IMS-assigned sequential Participant Identification Number (PID)	#####
SID	IMS-assigned Sample Identification Number (SID) (NIMH No. + Label No. per site)	###-####
PIN	IMS-assigned unique code for a PID (for security and external linkages)	####
Study Watch (aka wearable)	A Verily 16-alphanumeric serial number (manufactured device number)	A#AAA##A######AA
Neurocognitive Assessment	IMS-assigned PIN plus Group Number Assignment (i.e., 1, 2, 3, 4)	####-#
Digital Phenotyping	IMS-assigned username based on user-entered information at baseline	#######

The approach taken balances all factors to ensure linkage across the various data streams and the prospective nature of data collection.

The amount of beta-testing and system interconnection to ensure these seven linked IDs worked as specified and intended is intensive at start-up. Some unique challenges and our solutions are listed below:

- SID: Given the need to reduce study burden on busy ED staff, genomic labels were pre-printed for each ED site by participant and tube type. These pre-packed labels account for baseline and future biospecimens and were pre-loaded into the IMS to ensure each ED used labels only for the AURORA Study and only those for its site. Tailored quality control measures were included as part of the user interface to ensure protocol elements adhered to genomic standard operation procedures. The SID adhered to the National Institute of Mental Health (NIMH) dedicated repository laboratory information management system (LIMS) standards for specimen receipt, storage, linkage, and assay purposes. This was critical for the chain of custody that was IMS generated and controlled.
- PIN: We elected to assign an IMS PIN systematically for each participant identification (PID) as an extra security measure. The PIN is used on all communications (e.g., survey linkages, protocol reminders [emails and texts]) to narrow PID use on external linkages and as part of our tailored IMS security features.
- Study Watch: We used the Verily Life Sciences™ device as the Study Watch. The watch was provided to all participants at the time of enrollment. It captured continuous-time photoplethysmogram, 3-dimensional accelerometry, skin conductance, and environmental factors, including temperature, humidity, atmospheric/air pressure level, and ambient light; it also carried out on-demand electrocardiograms at various time points. De-identified and encrypted data were transmitted from the participant to the study team via a 3G or 4G LTE watch connectivity hub/charger provided to study participants [4]. “Study Watches: PID” are assigned “many-to-many” over the years but each device can only be assigned at one time to one PID.
- Neurocognitive Assessments: We used The Many Brains Project TestMyBrain.org™ platform to perform online cognitive tests. We used the PIN but supplemented with a Group Number for scientific purposes to randomize the order of the neurocognitive assessment batteries (NCAs) across participants in the repeated measurements. NCAs measure brain function, including reaction time, learning, concentration, personality, and memory. The study rotated balancing batteries of assessment across Weeks 1–8 and across the 3-, 6-, 9-, and 12-month follow-ups. NCAs take 10–15 minutes to complete, and participants received $20 for NCA1 completion and $5 for each subsequent NCA completion.
- Digital Phenotyping: We used the Mindstrong Health™ app to perform online “flash surveys” using mobile technology to measure brain function using participants’ designated smartphone. Flash Surveys are brief (about 5 minutes) updates using validated questions assessing peritraumatic, ongoing, and recovery time points. The surveys examine concepts like acute loss, depression, disorganization, self-regulation, panic attack, appetite disturbance, and sleep issues and problems. Incentives were paid ($1 per survey) for the completion of each of the 85 flash surveys administered. The app collected information on the following: (1) time and duration of phone calls; (2) time and number of texts sent and received; (3) keystroke information; (4) when swipes or taps were made on the phone; (5) when Wi-Fi access was being used; (6) a small percentage of the phone’s GPS location data; and (7) the number of times a word in a text was used over a 24-hour period. The app only collected the information that participants had consented to collect, never shared information with anyone not listed in their consent, used little battery, and did not interfere with phone performance.

### Operationalizing adaptive ED enrollment architecture

There are 13 steps for ED staff to conduct per enrolled participant (burden: approximately 3 hours of baseline data collection) while they are waiting to be treated for their index trauma. These activities are (1) screen for eligibility; (2) consent for all baseline items, including biological specimen collection; (3) enroll and install Mindstrong Health™ app for digital phenotyping; (4) assign a Study Watch and band for ecological monitoring; (5) conduct baseline ESense and ECG; (6) conduct ED Survey Part 1; (7) enroll and conduct TestMyBrain™ baseline NCA; (8) conduct ED Survey Part 2; (9) collect baseline blood biospecimens (and process and storage); (10) collect baseline saliva biospecimens (and process and storage); (11) pay baseline incentive; (12) review discharge and user credentials; and (13) medical record abstraction.

**Figure 1:**
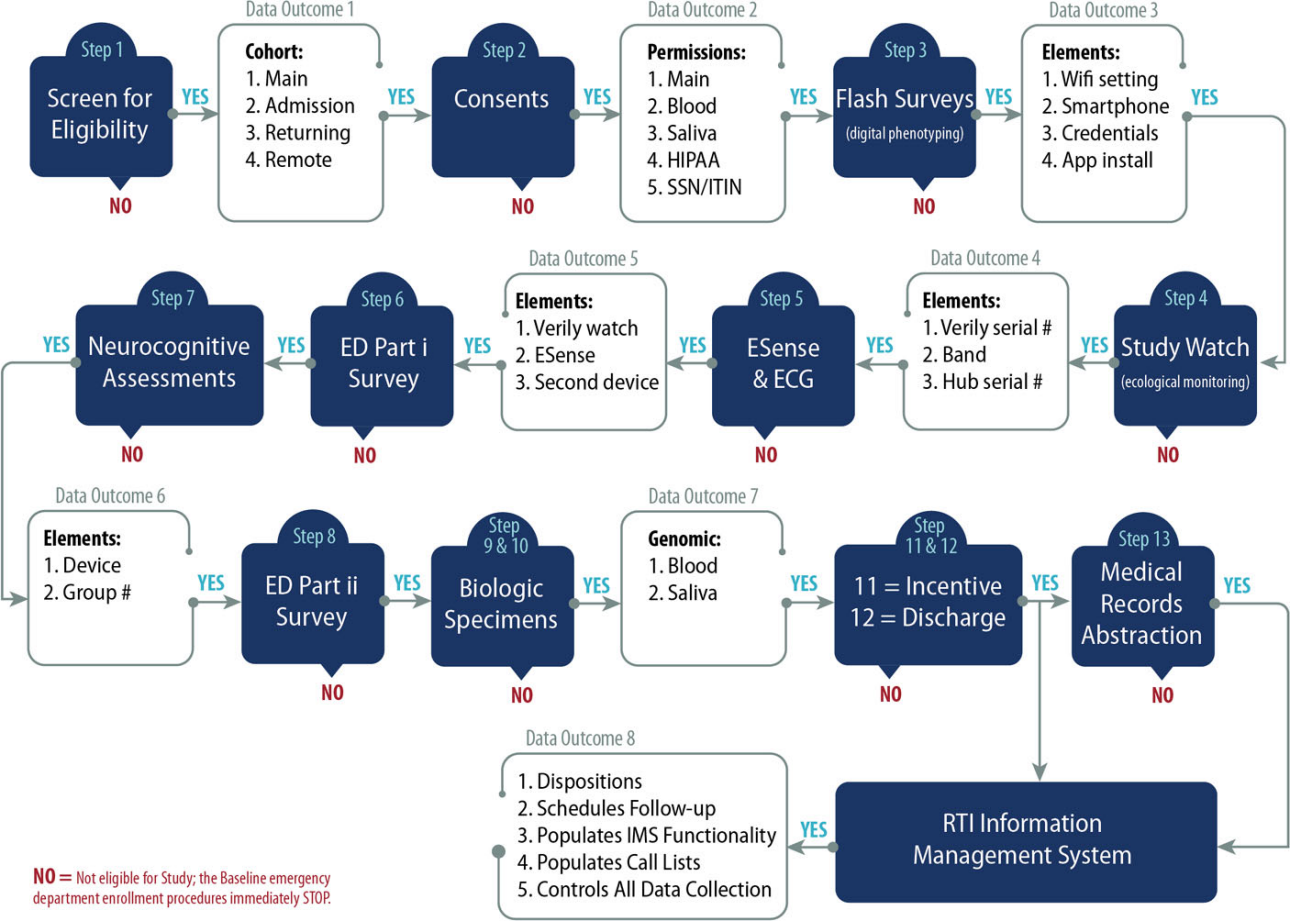
Baseline data streams. An integrated baseline program encompassing scripts, questions, graphics, instructions, and big data linkages to ease ED 3-hour protocol to incorporate phenotypic, clinical, physiological, sensor, environmental, ecological, and medical data measures

*Figure **1* shows a concatenated flow chart combining all steps and hundreds of pages of consents, scripts, questions, app and external system instructions and credentials, and study website usage into our single-use baseline program that encompasses the 3-hour baseline data collection effort. This enabled ED nurses and research coordinators to perform electronic baseline data collection on iPads via encrypted Wi-Fi technologies.

### Events & communications

To handle the numerous assessments per participant, there were up to 1250 possible events and protocol reminders. The participant contact protocol required sending emails and SMS messages by specific windows to invite/remind participants of their follow-up events. The IMS also tracked event completion to allow researchers to monitor cooperation and response rates. The IMS maintained blackout periods (i.e., periods during which the participant does not want to receive study communications), event histories, calling notes, incentive payment history and projected future payment dates. Finally, the system architecture had to be flexible to adapt to protocol changes in the multi-site and multi-year enrollment protocol to meet scientific objectives while adjusting to exogenous events such as the COVID-19 pandemic.

We adjusted the Nirvana backend study management system to make the web-based IMS completely data-driven for this protocol. Table 3 lists the approach used across the varied data sources. This data-driven master table enabled us to meet other system challenges including (1) participant preferences for contact dates/times; (2) need for both opening and closing dates for 1250 events/reminders; (3) need to use multiple communications channels (e.g., email, text/SMS, phone); (4) participants’ blackout periods/hours; (5) event statuses; (6) overall participant status (e.g., active, inactive, withdrawn, deceased); (7) incentive schema; and (8) allowance for updating per protocol modifications.

**Table 3:** Data-driven events and individual participant schedules

Event name	Day offset	Last day	Hours after offset	Event type ID	Via text	Via email	Via phone	Active	Incentive points	Complete status	Assessment ID	Survey ID
NCA1	1	6	0	1	NULL	NULL	NULL	1	400	2690	1	NULL
NCA1 Invite (Email)	1	1	2	2	0	1	0	1	0	1052	NULL	NULL
NCA1 Invite (Text)	1	1	2	2	1	0	0	1	0	1052	NULL	NULL
NCA1 Reminder (Email)	2	2	2	2	0	1	0	1	0	1052	NULL	NULL
NCA1 Reminder (Text)	2	2	2	2	1	0	0	1	0	1052	NULL	NULL
NCA1 Reminder (Email)	4	4	6	2	0	1	0	1	0	1052	NULL	NULL
8 Week Follow-Up	49	69	0	1	NULL	NULL	NULL	1	800	2690	NULL	1253
8 Week Follow-Up Invite (Email)	49	49	2	2	0	1	0	1	0	1052	NULL	NULL
8 Week Follow-Up Invite (Text)	49	49	2	2	1	0	0	1	0	1052	NULL	NULL
8 Week Follow-Up Reminder (Text)	49	49	2	2	1	0	0	0	0	1052	NULL	NULL

The approach taken to create 5000 individual schedules tied to the protocol events using enrollment date to populate 1250 events and reminders per participant.

### Blackout periods

To balance the scientific foci of AURORA and the goal of understanding, preventing, and predicting APNS conditions with the intensity of prospective data collections, it was part of the protocol to provide participants the ability to designate their preferred blackout days/times when the study would not perform automated communications (i.e., to help promote recovery). The following question was included in the initial baseline survey. An affirmative answer to this question was a requirement to participate in the AURORA Study; therefore, we have 100% compliance.

- *“We will be sending you notifications by text and email. Please tell us when you would prefer NOT to be contacted. For instance, when you are typically asleep. This can be up to a 10-hour period.”*

RTI’s IMS included data quality and consistency checks to ensure that a participant’s designated blackout period did not overlap with any of their best times to contact (i.e., to maximize compliance and response rates) previously collected in the same survey. Data collectors asked participants for clarification if there was overlap between these dichotomic periods. Table 4 summarizes the percentages of participants’ blackout periods for the $n=2641$ included in this analysis from September 2017 through February 2020. Over 93% of AURORA participants self-reported what appeared to be their sleeping hours as the blackout period.

**Table 4:** Participant blackout periods

	Females	Males
*Percentage of Participants with:*		
Defined Blackout Period	100%	100%
*Duration of Defined Blackout Period:*		
1 to 2 hours	0.56%	1.10%
3 to 5 hours	5.60%	5.54%
6 to 8 hours	26.59%	30.50%
9 to 10 hours	67.25%	62.86%

Solutions involved the creation of an Event Master Shell table that defines these 1250 events/reminders systematically: (1) event name/code; (2) day “offset” from Day 0 enrollment date; (3) last day; (4) hours from black out; (5) event type ID; (6–8) communication mode(s) (i.e., text, email, telephone) needed; (9) active status; (10) incentive points; (11) complete status; (12) assessment identification; and (13) survey identification.

## Methods

In addition to the previously defined schemas (i.e., identification linkage, data collection protocol specifications), creating a data-driven architecture utilizing such diverse data streams required pending and final disposition codes at each hand-off point to drive the next valid, acceptable, and logical data step. This required a mixture of experience, anticipated outcomes, and establishing an agile, flexible system to adopt to real-world evidence. We created 47 pending and 80 final status codes to drive this complex, adaptive IMS.

Another important consideration is the ability to track changes and allow for user error given the prospective nature and magnitude of data collection and the multiple staff interactions with the anticipated 5000 participants. We created a sophisticated SQL process to create a fully automatic audit file that tracks changes to all data fields (i.e., username, date/time stamp of change, original value, and new value). Staff and system users were aware of this monitoring and tracking system feature.

Although the number of system environments to develop, host, test, deploy, and maintain such an integrated, web-based, and adaptive IMS was typical of RTI’s diverse experience, we added a unique environment to handle the diversity and magnitude of the AURORA data that flowed daily. In addition to the standard system environments (i.e., development, stage, test, and production), we created a unique “Study Production” environment that used the same database structure and warehouse as the production environment but segmented the data by a single indicator that only RTI can control. Segmentation was valuable given the magnitude of the data sources, data fields, and the complexities of the adaptive sampling schema, routines, and data linkages. We can “test” the production environment in our HIPAA environment.

Given the burden on respondents of participating in up to 1250 events/reminders, we included a data-driven incentive schema that was updated in real-time and daily for the external big data (i.e., Study Watch, NCAs, flash surveys) events that are completed. As noted in the Regulatory section, the external DUAs were complex. As such, RTI decided not to embed full Application Programming Interfaces (APIs) to these external partners’ systems but link appropriate key variables to launch, monitor completion status, and securely exchange data each night via secure, batch jobs. This enabled each partner to minimize “system boundary” access issues via internal security policies and procedures. Therefore, participants could see daily how many incentive points had been earned and would be paid at the next incentive payment date. This data-driven adaptive schema played a crucial role in helping maintain effective compliance and response rates over the year-long participation period.

Another unique and adaptive IMS feature included site and user roles and responsibilities tailored to right-to-know and right-to-view capabilities to make the IMS user-friendly and only provide access to screens, data, and system functions germane to assigned tasks. For example, laboratory staff could only view biorepository screens when blood and/or saliva has been collected and shipped to the appropriate lab. After immediate processing of data at the next step, data became viewable only unless there was a need for editing and updating for future protocol purposes (see audit tracking above).

## Results

After balancing scientific trade-offs for biobehavioral, physiologic, and genomic outcomes versus big data acceptance and compliance, what do the interim results show? First, Table 5 shows the interim demographics of the trauma screened and/or population enrolled from September 2017 through March 2020. Slightly over 19% of approached EDs patients were eligible for participation. Recruitment continued but was heavily impacted by COVID-19 starting in March 2020. AURORA continues prospective follow-up of participants at least through early 2021.

**Table 5:** Interim demographics and eligibility

Demographics^1^	Female, age groups (%)^2^				Male, age groups (%)^2^			
	18–29	30–49	50–75	Subtotal	18–29	30–49	50–75	Subtotal
*Race*^*3*^ *(subtotal by Sex)*	**37%**	**41%**	**20%**	**100%**	**35%**	**45%**	**20%**	**101%**
White	13%	14%	8%	35%	13%	16%	9%	38%
African American	19%	23%	11%	52%	17%	22%	9%	48%
Asian American	1%	1%	–	2%	1%	1%	–	2%
Native Hawaiian or Pacific Islander	–	–	–	1%	–	–	–	1%
American Indian or Alaskan Native	1%	–	–	3%	1%	2%	1%	3%
Any Other Race(s)	3%	3%	1%	7%	3%	4%	1%	9%
*Ethnicity*^*4*^ *(subtotal by Sex)*	**37%**	**42%**	**20%**	**99%**	**34%**	**45%**	**20%**	**100%**
Not Hispanic or Latino	32%	38%	19%	89%	29%	39%	19%	87%
Hispanic or Latino	5%	4%	1%	10%	5%	6%	1%	13%
*Ineligibility*^*5*^ *(big data criteria only) (subtotal by Sex)*	**39%**	**38%**	**26%**	**99%**	**39%**	**35%**	**30%**	**101%**
– Refuses Study Watch	–	–	–	–	–	–	–	–
– Nickel Allergy	4%	4%	3%	10%	1%	1%	–	3%
– No Email Address	3%	5%	5%	13%	4%	6%	9%	20%
– Refuses Smartphone/Passive	3%	3%	2%	8%	12%	2%	2%	7%
– Physical Impairments re: Smartphone	4%	4%	3%	10%	–	6%	3%	13%
– No Smartphone or Will Not Use One	10%	10%	10%	29%	12%	10%	13%	35%
– Smartphone Use Less Than 1 Year	15%	12%	3%	29%	10%	10%	3%	23%
*Trauma Type*^*4*^ *(subtotal by Sex)*	**35%**	**38%**	**24%**	**99%**	**31%**	**38%**	**29%**	**98%**
Motor Vehicle Crash	23%	25%	14%	62%	16%	20%	13%	49%
Non-Motorized Collision	–	–	–	1%	1%	–	1%	2%
Physical Assault	7%	7%	2%	16%	7%	9%	5%	22%
Sexual Assault	1%	1%	–	2%	–	–	–	–
Fall, ≥10 Feet	–	1%	–	1%	1%	2%	1%	3%
Fall, <10 Feet	1%	2%	6%	9%	1%	–	4%	7%
Burns	–	–	–	1%	–	1%	1%	2%
Animal-Related	1%	1%	1%	3%	1%	1%	1%	2%
Other	2%	1%	1%	4%	4%	5%	3%	11%

1. An overview of the eligibility of the trauma patients screened for this study. 2. Percentages listed use sex as the denominator and exclude “missing” data and are subject to rounding. 3. Race uses the denominator of *n* = 2641 participants screened, whether eligible or ineligible, who also provided their age. 4. Ethnicity and Trauma Type use the denominator of *n* = 2500 participants screened, whether eligible or ineligible, who also provided their age. 5. Ineligibility uses the denominator of *n* = 1174 participants screened ineligible for one of these seven “big data” exclusion criteria who also provided their age.

**Figure 2:**
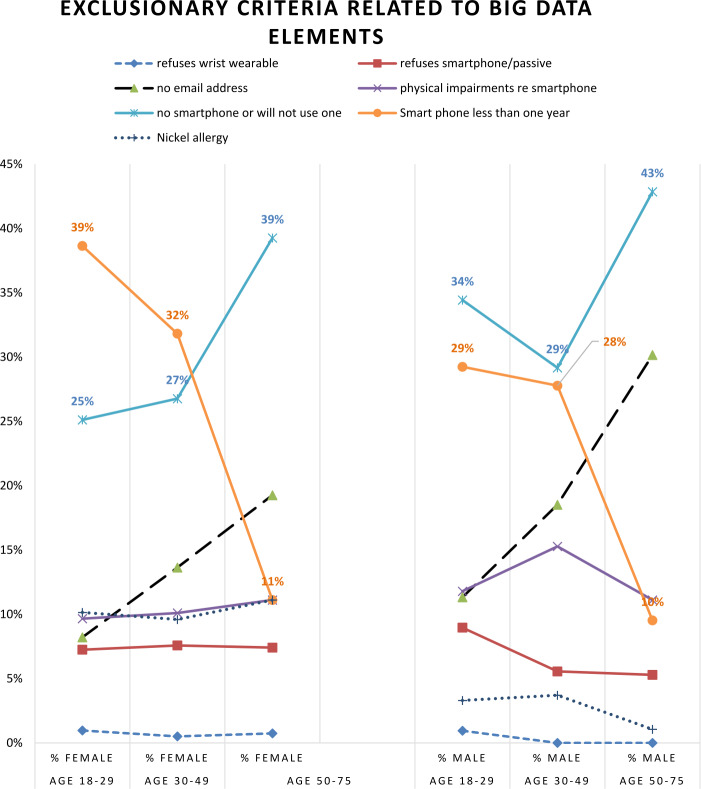
Exclusionary criteria related to big data items. The seven eligibility criteria related to ecological/passive, digital phenotyping, and online neurocognitive assessments are noted. The “ineligibility” denominator consists of $n=1174$ participants screened ineligible for one of these seven “big data” exclusion criteria who also provided their age

Figure 2 shows the ineligibility, stratified by sex and age categories, of trauma patients screened from September 2017 through February 2020 concerning the “big data” eligibility criteria. Although only approximately 14% refused these three “big data” data collections, there is varied eligibility by sex and age categories. On average, men were less eligible than women, and as expected, the older age group was less likely to have/use a smartphone or have an email address.

**Figure 3:**
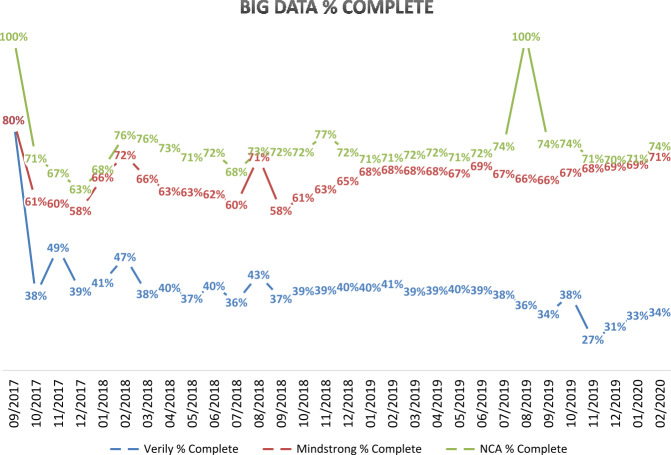
Compliance to wearable, flash surveys/digital phenotyping, and neurocognitive assessments. Interim compliance related to ecological/passive, digital phenotyping, and online neurocognitive assessments are noted

Figure 3 shows the percent of completed Study Watch, flash surveys, and NCAs from September 2017 through March 2020. We considered all participants who reached these data collection event milestones (i.e., open date occurred and the participants were actively enrolled) and classified whether they (1) completed that event or (2) the window expired without data. Participant compliance, from high to low, were NCAs, flash surveys, and Study Watch.

## Conclusion

A web-based, data-driven, integrated, and adaptive health IMS that connects, links, and enriches diverse data streams is possible, but the degree of design, testing, and systematic enhancements should be carefully considered, planned, and thoroughly tested. The degree of complex testing this requires is considerable and rivals the magnitude of data that are exchanged daily. For example, every day we transfer over 62 billion data points with the UNC School of Medicine. Automated ingestion, processing, quality control, storage, and delivery routines are paramount given the magnitude of data exchange. The hourly ($n=23$ separate routines) and daily batch jobs are critical to updating pending and final status disposition codes and enabling views of progress per participant across all data streams.

The key lessons learned when linking various sources of big data (i.e., wearable technology, physiological, ecological monitoring, genomic, neurocognitive assessments, brain imaging, and psychophysical) with survey data are listed in Table 6.

**Table 6:** Lesson learned when connecting, enriching, and linking “big data” with survey data

Research challenge	Solution(s) or idea(s)
Regulatory requirements	• Involve legal, contracts, and procurements staff early in the process • Be prepared to adjust system architecture and computing center environmental specifications • Take advantage of U.S. funder (NIH) policies such as single IRB and Certificates of Confidentiality • Adjust quality control processes and procedures for monitoring, reporting, and auditing • Be prepared to limit data storage to select partners and data warehouses per DUAs • Negotiate onerous terms and conditions that do not adhere to study design and Prime contractual requirements
Protocol complexities	• Create an IMS that ∘ Is data-driven ∘ Is web-based ∘ Enables adaptive sampling ∘ Uses multiple linking identification schemas ∘ Uses an agile approach that ensures participants and staff find the IMS user-friendly ∘ Allows tailored event, incentive, reporting, and delivery routines and functionality ∘ Is scalable and flexible enough to accommodate protocol modifications ∘ Adheres to security, privacy, and compliance requirements
Multiple technology partners and/or platforms	• Plan for differential DUAs and accompanying limitations and understand the implications for the protocol and architecture (e.g., monitoring, security reviews, level of access) • Negotiate system boundaries by partner and platform; adjust if (and level of) system interconnections necessary to accomplish re linkage and data capture, status reporting, and delivery • Budget appropriately to account for software, platform, and technological enhancements (e.g., new versions, downtimes, security changes) and how e-linkages between systems are affected (scalability and flexibility are key constructs) • Ensure specific hardware/sensors, manufacturing and production schedules can achieve main protocol recruitment timelines, sharing/distribution of sensors, and implications on chain of custody and analysis • Plan that participating laboratories often have existing LIMS with unique data linking requirements and may be outdated and separate from laboratory-to-laboratory

Key research challenges and solutions and ideas we found useful when building such complex information management systems.

In summary, the regulatory requirement solutions balanced study design (observational, not a clinical trial); security and privacy (e.g., HIPAA, BAA, system access and boundaries); human subjects/IRB; and other study agreements such as DUAs between parties (e.g., auditing, monitoring). Because RTI hosted and housed AURORA’s personally identifiable information/protected health information within our secure, NIH-compliant computing center, we balanced competing and diverse regulatory requirements via a tiered approach and adherence to (1) our cooperative agreement terms and conditions with the Prime contractor; (2) NIH and Prime security and IRB policies; (3) RTI’s access, security, and monitoring policies; and (4) any third-party DUAs. This was a deliberate tiered approach because we did not accept lower rated requirements that conflicted with higher priority requirements. We negotiated such conflicts accordingly and did not sacrifice overall IMS functionality, security, auditing, and monitoring requirements.

The critical lesson learned for the complex protocol requirements was to create, maintain, and update complete and thorough system specifications throughout the study lifecycle. RTI used a systematic change control process for each of the $n=37$ production IMS updates as of December 2020. This is critically important as RTI requires all participating sites to simultaneously use the “same production version” across the $n=87$ survey versions implemented thus far in terms of data integrity and consistency. Each fully documented change control process update is treated as a deliverable with unique “versioning numbering schema” in place across all data streams. This is critical and invaluable for data analytical purposes given the complexity of the protocol and diverse data sources.

The overarching theme of utilizing various technologies and platforms was for RTI’s team to simplify and create a seamless, integrated experience for system users (i.e., participants, ED coordinators and staff, follow-up personnel, laboratory staff, principal investigators, and RTI staff). This is easier stated than it is to accomplish, and the process of using multiple technologies and platforms should not be underestimated and budgeted. As the creator and host of the primary platform/IMS, our team had to adjust to the challenges and limitations of partner organizations’ technology and platforms more than we anticipated. We used the regulatory requirement tiered approach described above to frame our statement of work and adjusted the budget resources accordingly to account for Verily Study Watch, TestMyBrain neurocognitive assessments, and Mindstrong Health digital phenotyping big data technologies, platforms, and architecture needs. This flexibility and scalability also applied to the participating genomic laboratories to maintain linkage and biospecimen chain of custody.

Another lesson learned is the need to design and account for some participants electing to continue using external apps after their study involvement. Participants may decide they like using one or more study apps or platforms and continue to use them after study involvement. Therefore, it is important to designate a permanent study close date (i.e., from Day 0) to disenable post-study data flows into the data warehouse.

In the AURORA Study, we had low ineligibility tagged to the seven big data inclusion criteria; men were less eligible than women across all age categories. Refusals to the big data protocol elements were mixed across sex/age strata. We had few trauma participants refuse to receive the Study Watch; however, 10% of the women were allergic to nickel and therefore ineligible for ecological monitoring and the study. In this target population, participants were most compliant with NCAs, followed by flash surveys, and then the Study Watch.

Conducting complex biomedical research that encompasses, enriches, and links neurocognitive, digital phenotyping, physiologic, psychophysical, neuroimaging, genomic, and sensor data with survey data brings many system challenges but participants, research, and laboratory personnel quickly rise to meet these challenges. To accomplish such state-of-the-art assessments, IMSs should be data-driven, robust, and encompass all protocol and data collection elements in a sophisticated, user-friendly, and secure web-based environment.

## Data Availability

The datasets generated during and/or analyzed during the current study are not publicly available as the AURORA continues participant follow-up into at least 2021. The AURORA Study executive and investigative teams hopes to make the dataset available to the scientific community via the NIMH Data Archive.

## Abbreviations

APIs: Application Programming Interfaces
APNS: Adverse Posttraumatic Neuropsychiatric Sequelae
AURORA: Study Advancing Understanding of RecOvery afteR TraumA
BAA: Business Associate Agreements
DUAs: Data Use Agreements
ED: Emergency Department
HIPAA: The Health Insurance Portability and Accountability Act of 1996
IRB: Institutional Review Board
IMS: Information Management System
LIMS: Laboratory Information Management System
NCAs: Neurocognitive Assessment Batteries
NIH: The National Institutes of Health
NIMH: National Institute of Mental Health
PID: Participant Identification
PIN: Participant Identification Number
RTI: RTI International
SID: Sample Identification
UNC: The University of North Carolina at Chapel Hill
